# Supplementary figures and images for: Women are underrepresented in computational biology: An analysis of the scholarly literature in biology, computer science and computational biology
Source: PLoS Comput Biol. 2017 Oct 12;13(10):e1005134. doi: 10.1371/journal.pcbi.1005134 (PMC5638210; doi:10.1371/journal.pcbi.1005134)

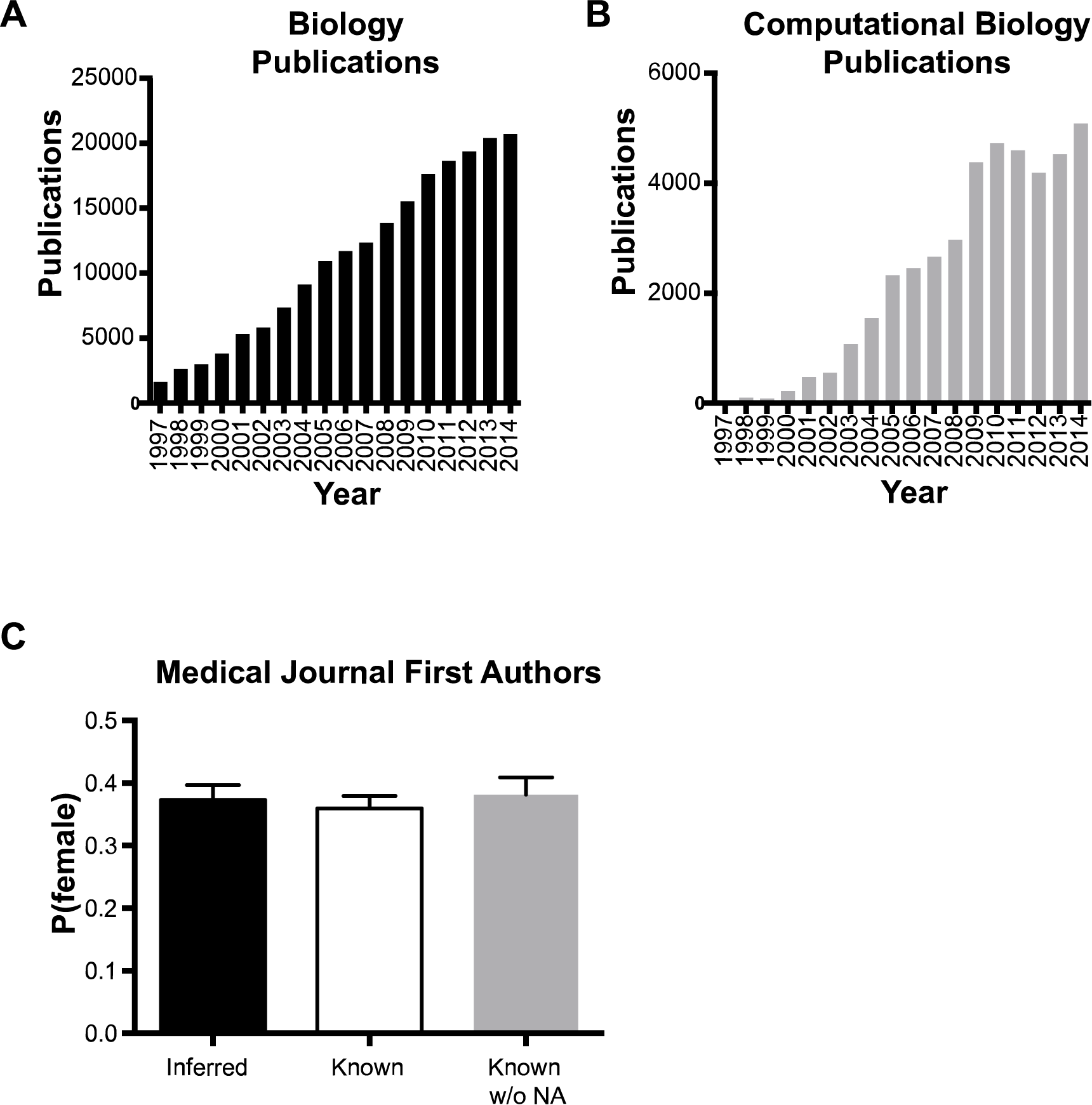

Supplement: S1 Fig — A: Number of primary publications per year indexed under the “Biology” MeSH term. B: Number of primary publications per year indexed with “Computational Biology” as a major MeSH term. C: Comparison of computational gender inference (black) with known genders (white) for the dataset from Filardo et. al. [13]. Grey represents the known proportion of female authors when excluding names for which the gender could not be computationally inferred. Error bars represent 95% confidence intervals. (TIF) [file pcbi.1005134.s001.tif]

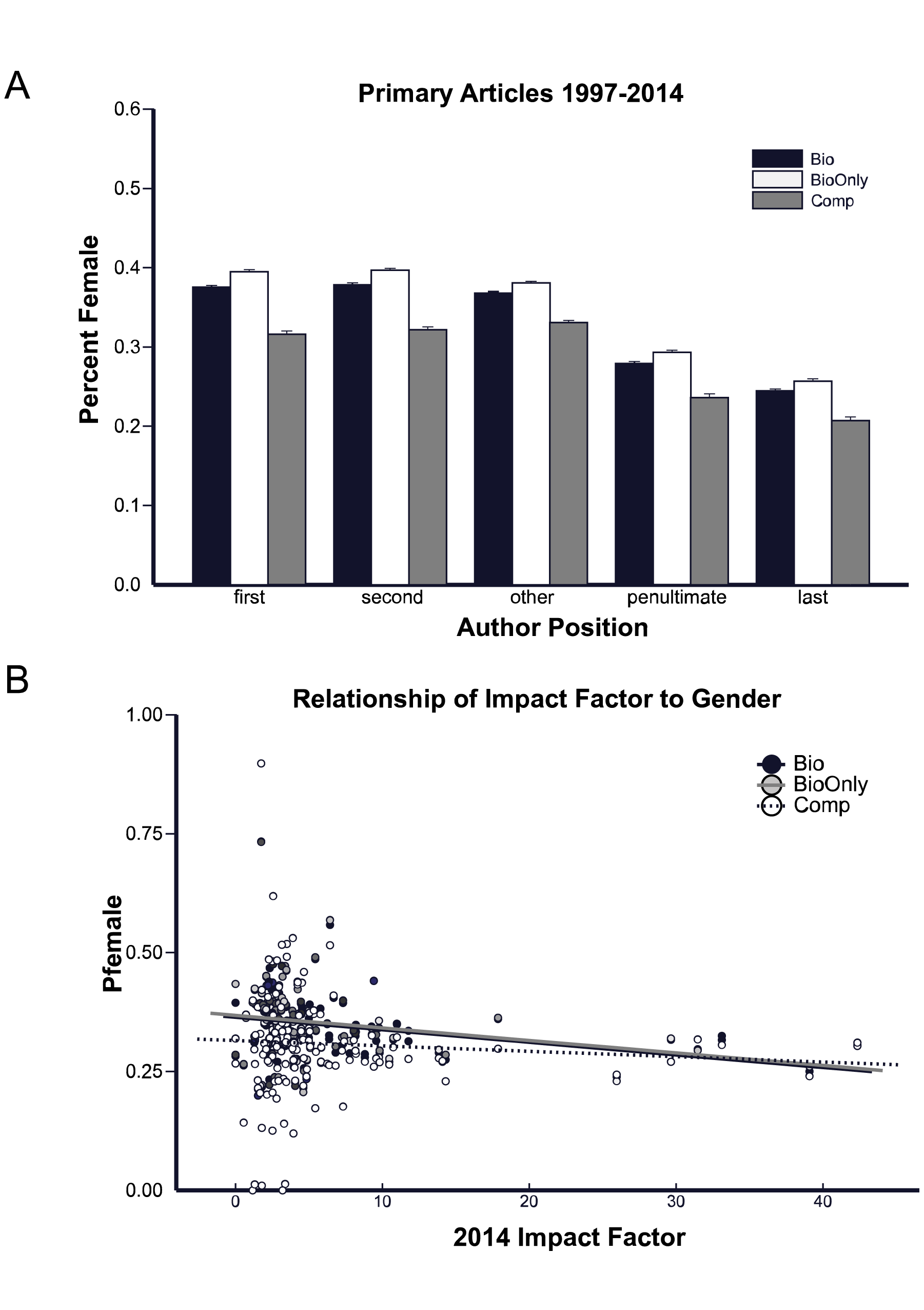

Supplement: S2 Fig — A: Mean probability that an author in a given position is female for primary articles indexed in Pubmed with the MeSH term Biology (black), Computational Biology (gray) or for those articles with Biology but not Computational biology (white). Error bars represent 95% confidence intervals. B: Mean probability that an author is female for articles in the “Bio” dataset (black) in the “Comp” dataset (white), or for articles in the Bio but not Comp (gray) for each journal that had at least 1000 authors plotted against the journals’ 2014 impact factor. Excluding computational publications from the biology dataset does not substantially alter the correlation between impact factor and Pfemale. (TIF) [file pcbi.1005134.s002.tif]
